# Outcomes of reoperative aortic valve surgery: data from a national registry over 23 years

**DOI:** 10.1007/s12055-025-01980-1

**Published:** 2025-06-17

**Authors:** Pradeep Narayan, Arnaldo Dimagli, Jeremy Chan, Tim Dong, Charles Tan, Tugba Aydin, Daniel Paul Fudulu, Gianni Davide Angelini

**Affiliations:** 1 Narayana Health, Bangalore, India; 2 Bristol Heart Institute, Bristol Royal Infirmary, Bristol University, Upper Maudlin Street, Bristol, BS2 8HW UK

**Keywords:** Re-operative surgery, Aortic valve replacement, Redo aortic valve surgery

## Abstract

**Objective:**

Over the past two decades, early mortality following re-operative aortic valve surgery has declined significantly; however, it remains higher than that observed after primary isolated valve replacement. We sought to examine temporal trends and identify independent predictors of adverse outcomes in patients undergoing re-operative aortic valve surgery.

**Method:**

The study included all patients undergoing re-operative aortic valve replacement (AVR) in the United Kingdom between January-1996 and March-2019 including those with multiple previous operations and those undergoing additional procedures. Data was obtained from the National Institute of Cardiovascular Outcomes Research database. The primary objective was to assess in-hospital mortality trends. Secondary objective was to identify risk factors for in-hospital mortality. Multivariable analysis was carried out to identify independent risk factors for in-hospital mortality.

**Results:**

During the study period, 6,109 re-operative aortic valve surgeries were carried out in the United Kingdom. There were 1,973(32%) females, median age was 69(60–76) years with median duration between the initial and the reoperation being 7(2–13) years. Bio-prosthetic valves were more commonly explanted compared to mechanical valves: 4,125(68%) vs. 1,641(27%). Mortality for elective re-operative cases was 4.8% (*n* = 166). After adjustments, surgery after 2007, age, number of previous operations, urgency of operation, gender, concomitant procedures, pre-operative chronic kidney disease and endocarditis, were important predictors of outcomes. Mortality showed a downward trend during the study period (*p* < 0.001).

**Conclusion:**

With advances in management strategies, mortality following re-operative AVR continues to decline, but still remains significant. Structural degeneration of bioprosthetic valves continues to be the most common indication for re-operation Emergency re-operations are associated with substantially higher mortality rates; therefore, close follow-up of these patients is essential to facilitate timely elective re-intervention before clinical deterioration.

**Graphical Abstract:**

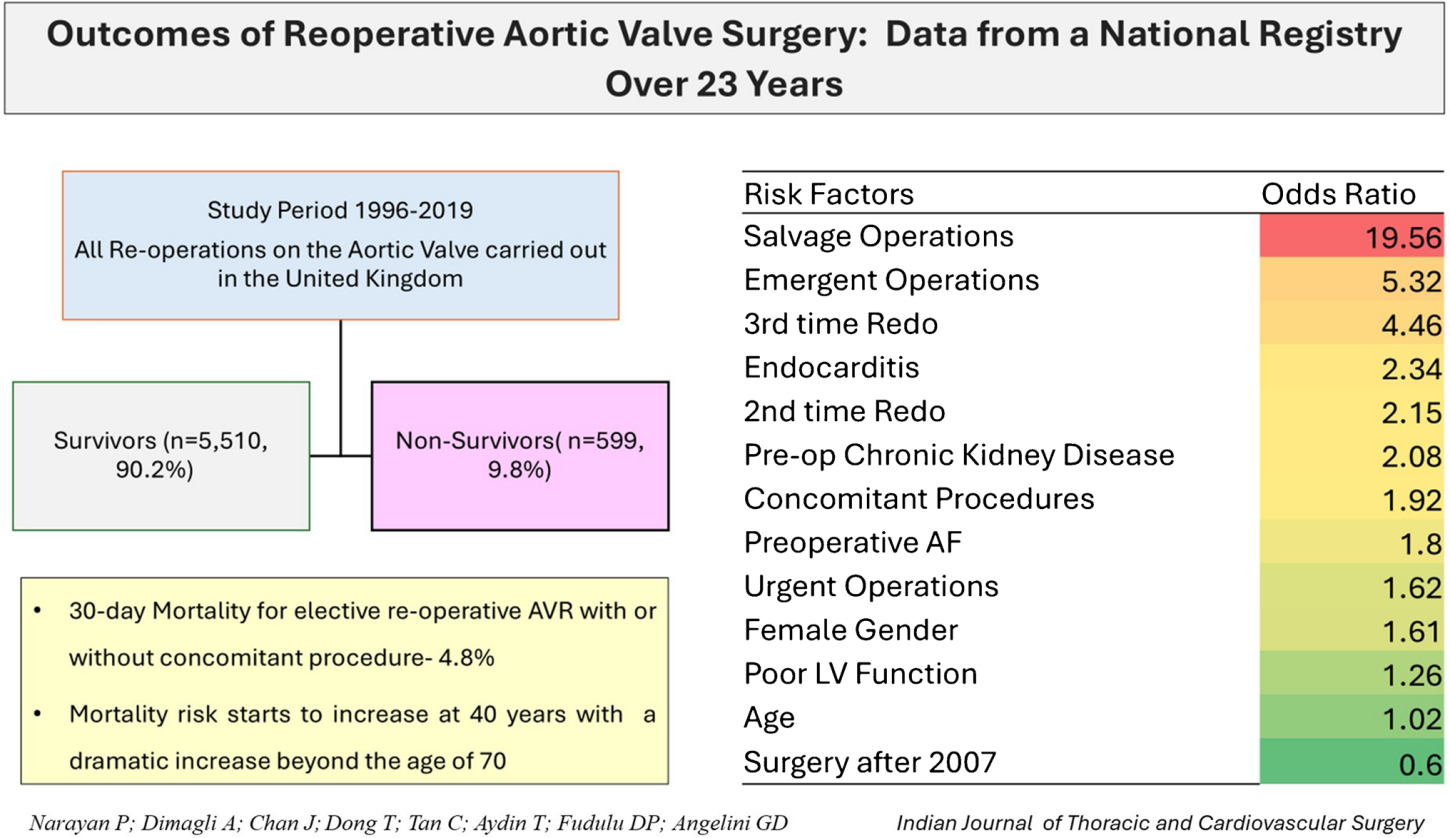

**Supplementary Information:**

The online version contains supplementary material available at 10.1007/s12055-025-01980-1.

## Introduction

Reoperations on the aortic valve may be indicated for multiple reasons and include structural and non-structural dysfunctions. Structural valve degeneration of bioprosthetic valves constitutes the most common indication for re-operative aortic valve surgery [1, 2]. Some of the other common indications include aortic valve endocarditis [3–6], valve thrombosis [6], severe para-prosthetic leaks [6], severe patient prosthesis mismatch [5, 6] and valve malfunction due to pannus formation [6]. All these pathologies result in a valve that does not provide the expected hemodynamic performance and thus constitutes an indication for reoperation.

The increasing adoption of bioprosthetic valves over the past few decades has significantly reshaped the landscape of aortic valve reoperations. An analysis of the Society of Thoracic Surgeons database revealed that bioprosthetic valve implantation increased from 34% in 1988 to 75% in 2021 [1, 7]. A similar trend was observed in data from the Society for Cardiothoracic Surgery in Great Britain and Ireland, reflecting a global shift toward bioprosthetic valves [8]. The advent of Transcatheter Aortic Valve Replacement (TAVR) has further influenced this shift, with the implantation of mechanical valves decreasing from 24.8% in the pre-TAVR era to a mere 12.2% after the advent of TAVR, which has superseded surgical aortic valve replacement (SAVR) in some countries and is emerging as another important indication for re-operative surgery [9–11].

Early mortality and postoperative morbidity in re-operative aortic valve surgery continues to decline but still remains high compared to primary isolated aortic valve replacement (AVR) [12]. While the mortality in the late sixties and early seventies was 41%, it reduced to 11% during the nineties, with contemporary literature reporting mortality between 4–6% [1, 5, 12–15].

Multiple factors influence surgical outcomes beyond temporal trends, including the urgency of operation, need for concomitant procedures, and type of explanted valve [3, 15–18]. Despite numerous studies examining reoperative aortic valve surgery, existing literature is limited by small sample sizes and predominantly consists of single-institution experiences. There remains a critical need to understand contemporary mortality trends and identify specific risk factors in the current era, particularly given the evolving surgical techniques and changing patient populations. In this study, we sought to analyse mortality trends and identify independent predictors of adverse outcomes using nationwide data, representing one of the largest reported cohorts of patients undergoing reoperative aortic valve surgery. This study includes patients who underwent multiple prior aortic valve procedures as well as those who had concomitant procedures at the time of redo surgery. By capturing this broader cohort, we also aim to provide a comprehensive understanding of the complexity and outcomes of redo aortic valve surgery in a real-world setting, rather than presenting an overly selective analysis limited to isolated reoperative AVR.

## Material and methods

The study included all patients undergoing re-operative aortic valve surgery in the United Kingdom between January 1996—March 2019. Data were obtained from the National Institute of Cardiovascular Outcomes Research (NICOR) central cardiac database, which collects data in a prospective fashion as part of the National Adult Cardiac Surgery Audit (NACSA). The NICOR registry collected demographic, intra-operative and postoperative mortality and morbidity data, for all adult cardiac surgery procedures performed in the United Kingdom (UK). Detailed process flow from input to analysis has been described before [19].

The study is part of a research project approved by the Health Research Authority (HRA) and Health and Care Research Wales(HCRW). As the study included retrospective interrogation of the NICOR database the need for individual patient consent was waived off (HCRW Integrated Research Application System ID: 278171) in accordance with the research guidance. The study was performed in accordance with the ethical standards as laid down in the 1964 Declaration of Helsinki and its later amendments.

### Data extraction and cleaning

A retrospective analysis was carried out on the complete dataset data collected prospectively as part of the NACSA. Briefly, data was entered by surgeons and validated at the local level by database managers and then uploaded through a web-portal to NICOR. Further validation was performed according to logical rules, and data reports were generated for missing primary variables. The academic healthcare informatics department then carried out the data cleaning. Duplicate records were removed, transcriptional discrepancies re-coded and clinical and temporal conflicts resolved. Missing data were resolved during the validation stages of the data transfer from individual centers. Missing and conflicting data for in-hospital mortality status were backfilled and validated via record linkage to the Office for National Statistics (ONS) census database. Missing data for baseline information in the NICOR registry was very low (1.7%). Missing categorical or dichotomous variable data were imputed with the mode while missing continuous variables data was imputed with the median.

The primary objective was to assess in-hospital mortality trends. Secondary objective was to identify risk factors for in-hospital mortality. Urgent surgery was defined as non-elective admission, with surgery being carried out during the same admission. Emergency procedure was defined as an operation carried out on the same day, and salvage procedure was defined as patients requiring cardiopulmonary resuscitation en route to the operating room or before induction of anesthesia. Poor left ventricular (LV) ejection fraction was defined as LVEF < 30% and very poor ejection fraction as LVEF ≤ 20%.

### Statistical methods

Continuous variables were assessed for normality using the Shapiro–Wilk test and visual inspection of histograms. Due to non-normal distribution of several key variables, continuous data are presented as median and interquartile range (IQR), and the Wilcoxon rank-sum test was used for comparisons. Categorical variables are presented as count and proportion and were compared using the Pearson's Chi-squared test or Fisher's exact test, as appropriate.

Patients’ characteristics were reported in the overall sample and were stratified by patient status at discharge (survivor vs non-survivors). Predictors of operative mortality were investigated using a Multiple Logistic Regression model, which included the baseline patient characteristics found significant at the univariable analysis. The forward stepwise selection of independent variables from the list above for inclusion in the final model was guided by the Akaike Information Criterion, employing 500 bootstrap datasets [20]. Variables that exhibited statistical significance in at least 50% of the iterations were retained in the final model. A restricted cubic spline curve of age vs. mortality adjusted for the significant variables detected was then plotted.

As surgical outcomes may have improved in the last decade, we investigated the outcome dividing the 23-year timeframe into two eras: 1996–2007 and 2008–2018. Effect estimates were reported as odds ratio (OR) and 95% confidence interval (CI). *P*-value < 0.05 was considered significant in all the analysis. Statistical analysis was performed using R version 4.0.0.

## Results

During the study period, 6,109 re-operative aortic valve surgeries were carried out in the United Kingdom. There were 1,973(32%) females, and the median age of the entire cohort was 69(60–76) years. The median duration between the first and the second operation was 7(2–13) years. In our cohort, the main indications for reoperation were structural valve degeneration (2,921 patients, 47.8%), endocarditis (1,587 patients, 25.9%), and non-structural valve dysfunction (1,601 patients, 26.2%). Six hundred and one (9.8%) patients had a history of previous myocardial infarctions. 432 (7.1%) required emergency surgery, and 69 (1.2%) needed re-operative surgery as a salvage procedure. 167 (2.7%) patients presented with cardiogenic shock before the procedure. Bio-prosthetic valves were more commonly explanted 4,125 (68%). Bio-prosthetic valves were also implanted in the majority [3,908 (64%)] of the patients at the time of re-operative surgery. 

Among the cohort, 2,113 (34.6%) of the patients required one or more concomitant procedures, with Coronary Artery Bypass Grafting (CABG) carried out in 1,570 (26%) patients. Majority of the patients had a single valve replacement, whereas 636 (11%) had a double valve replacement, and 92 (1.5%) had a triple valve replacement. Median EuroSCORE II was 6 (4–11).

The groups were divided into survivors (*n* = 5,510) and non-survivors (599). There was a similar number of female patients in the two groups. The incidence of diabetes, hypertension, history of smoking, previous neurological event, and left main stem stenosis presence was similar across the two groups (Table 1). Non-survivors had a significantly higher EuroSCORE II with a median of 13 (7–25) compared to survivors who had a median EuroSCORE II of 6 (3-10), *p* < 0.001 and a history of previous myocardial infarction (MI) in a significantly higher number of patients (*p* < 0.001). A significantly larger proportion of non-survivors had severely impaired LV function compared to survivors (44% vs. 30%, *p* < 0.001). Endocarditis was significantly more common among non-survivors (299 patients, 49.9%) compared to survivors (1,288 patients, 23.4%). Non-survivors also presented with more severe conditions, with a higher number requiring surgery under critical circumstances—either as a salvage procedure (5.8% vs. 0.61%) or as an emergency (23% vs. 5.4%) procedure. Cardiogenic shock was present in 11% (63) of non-survivors compared with 1.9% (104) of survivors. Non-survivors also had a much larger proportion of patients requiring ventilation prior to the surgery [58 (9.7%) vs. 90 (1.6%) in survivors (Table 1).

**Table 1:** Baseline characteristics

	Overall *n* = 6,109	Survivors *n* = 5,510	Non-survivors *n* = 599	*p*-value
Age (yrs)	69 (60, 76)	69 (59, 76)	71 (61, 76)	0.003
Females	1,973 (32%)	1,772 (32%)	201 (34%)	0.5
EuroSCORE II	6 (4, 11)	6 (3, 10)	13 (7, 25)	< 0.001
BMI (kg/m^2^)	26.9 (24.2, 30.1)	26.9 (24.3, 30.1)	26.8 (23.5, 29.3)	0.003
Previous MI	601 (9.8%)	515 (9.3%)	86 (14%)	< 0.001
Carotid Endarterectomy	5 (< 0.1%)	3 (< 0.1%)	2 (0.3%)	0.079
No of Previous operations				< 0.001
One	5,506 (90%)	5,043 (92%)	463 (77%)	
Two	570 (9.3%)	444 (8.1%)	126 (21%)	
Three	33 (0.5%)	23 (0.4%)	10 (1.7%)	
Duration between re-operation (yrs)	7 (2, 13)	7 (2, 13)	4 (1, 10)	< 0.001
Diabetes				0.13
Diet controlled	193 (3.2%)	172 (3.1%)	21 (3.5%)	
On oral hypoglycemics	527 (8.6%)	478 (8.7%)	49 (8.2%)	
Insulin dependent	191 (3.1%)	163 (3.0%)	28 (4.7%)	
Hypertension	3,481 (57%)	3,144 (57%)	337 (56%)	0.7
Smoking				0.6
Never smoked	2,742 (45%)	2,476 (45%)	266 (44%)	
Ex-smoker	2,920 (48%)	2,625 (48%)	295 (49%)	
Current smoker	447 (7.3%)	409 (7.4%)	38 (6.3%)	
Pre-op Chronic Kidney Disease	338 (5.5%)	236 (4.3%)	102 (17%)	< 0.001
Endocarditis	1587(25.9%)	1288(23.4%)	299(49.9%)	< 0.001
Pulmonary Disease	819 (13%)	722 (13%)	97 (16%)	0.035
Pre-operative CVA	675 (11%)	597 (11%)	78 (13%)	0.10
Peripheral Vascular Disease	528 (8.6%)	444 (8.1%)	84 (14%)	< 0.001
Pre-op Atrial Fibrillation	874 (14%)	748 (14%)	126 (21%)	< 0.001
Pre-op ventricular arrhythmias	15 (0.2%)	10 (0.2%)	5 (0.8%)	0.012
Pre-operative pacing	288 (4.7%)	227 (4.1%)	61 (10%)	< 0.001
Left Main Stem Stenosis	15 (0.2%)	12 (0.2%)	3 (0.5%)	0.2
Left Ventricular EF				< 0.001
Very severe impairment	8 (0.1%)	7 (0.1%)	1 (0.2%)	
Severe impairment	1,943 (32%)	1,680 (30%)	263 (44%)	
Moderate impairment	121 (2.0%)	111 (2.0%)	10 (1.7%)	
No impairment	4,037 (66%)	3,712 (67%)	325 (54%)	
Pulmonary Hypertension	185 (3.0%)	140 (2.5%)	45 (7.5%)	< 0.001
Cardiogenic Shock	167 (2.7%)	104 (1.9%)	63 (11%)	< 0.001
Pre-op Inotropes	278 (4.6%)	193 (3.5%)	85 (14%)	< 0.001
Pre-op Ventilation	148 (2.4%)	90 (1.6%)	58 (9.7%)	< 0.001
Urgency				< 0.001
Elective	3,400 (56%)	3,234 (59%)	166 (28%)	
Urgent	2,208 (36%)	1,947 (35%)	261 (44%)	
Emergency	432 (7.1%)	295 (5.4%)	137 (23%)	
Salvage	69 (1.2%)	34 (0.61%)	35(5.8%)	

*BMI* Body Mass Index, *MI* Myocardial Infarction, *CVA* Cerebro-Vascular Accidents, *EF* Ejection Fraction

A regurgitant lesion was significantly more common among non-survivors (303 patients, 50.5%) compared to survivors (2,046 patients, 37%), whereas a stenotic lesion was more frequently observed among survivors (2,279 patients, 41.3%) than non-survivors (142 patients, 23.7%). In 189 (32%) patients in the non-survivor group, the explanted valve was mechanical as opposed to 1,452 (26%) among the survivors. The complexity of procedures among non-survivors was significantly higher, with 36% of non-survivors needing one additional procedure, 7.7% needing two, and 1% needing three additional procedures compared to 30%, 2.8%, and 0.4% respectively in the group that survived. Concomitant mitral valve procedures were carried out in 624(10.1%) patients with 444 (71.1%) undergoing mitral valve replacements and 180 (28.2%) undergoing mitral valve repairs. Concomitant mitral valve procedures were carried out among 526 (9.5%) of the survivors compared to 98 (16.3%) of the non-survivors, *p* < 0.001. Concomitant CABG was significantly more common among non-survivors (193 patients, 32%) compared to survivors (1,377 patients, 25%; *p* < 0.001) (Table 2).

**Table 2:** Intra-operative variables

	Overall *n* = 6,109	Survivors *n* = 5,510	Non-survivors *n* = 599	*p*-value
Concomitant procedures				< 0.001
Isolated	3,996 (65%)	3,662 (66%)	334 (56%)	
One-additional procedure	1,884 (31%)	1,671 (30%)	213 (36%)	
Two-additional procedures	202 (3.3%)	156 (2.8%)	46 (7.7%)	
Three-additional procedures	27 (0.4%)	21 (0.4%)	6 (1.0%)	
Median Sternotomy	6,093 (99.7%)	5,495 (99.7%)	598 (99.8%)	0.6
Partial Sternotomy	8 (0.1%)	8 (0.1%)	0 (0%)	> 0.9
Mini Thoracotomy	1 (< 0.1%)	1 (< 0.1%)	0 (0%)	> 0.9
Other Incision	7 (0.1%)	6 (0.1%)	1 (0.2%)	0.5
Type of explanted aortic valve				0.045
Mechanical	1,641 (27%)	1,452 (26%)	189 (32%)	
Biological	4,125 (68%)	3,745 (68%)	380 (63%)	
Homograft	300 (4.9%)	272 (4.9%)	28 (4.7%)	
Autograft	43 (0.7%)	41 (0.7%)	2 (0.3%)	
Type of Implanted aortic valve				< 0.001
Mechanical	1,692 (28%)	1,543 (28%)	149 (25%)	
Biological	3,908 (64%)	3,551 (64%)	357 (60%)	
Homograft	285 (4.8%)	213 (4.0%)	72 (12%)	
Concomitant CABG	1,570 (26%)	1,377 (25%)	193 (32%)	< 0.001
Number of Valves replaced				< 0.001
Single Valve	5,253 (85.9%)	4,808 (87.2%)	445 (74%)	
Double Valve	636 (11%)	519 (9.6%)	117 (20%)	
Triple valve	92 (1.5%)	67 (1.2%)	25 (4.3%)	
Aortic valve pathology				< 0.001
Stenosis	2,421 (39.6%)	2,279 (41.3%)	142 (23.7%)	
Regurgitation	2,349 (38.5%)	2,046 (37%)	303 (50.5%)	
Mixed	1,017 (16.7%)	921 (16.7%)	96 (16%)	
CPB (minutes)	127 (94, 182)	123 (92, 170)	223 (151, 317)	< 0.001
Cross-Clamp time (minutes)	90 (67, 127)	88 (66, 121)	127 (90, 181)	< 0.001

*CABG* Coronary Artery Bypass Grafting, *CPB* Cardiopulmonary Bypass

**Fig. 1:**
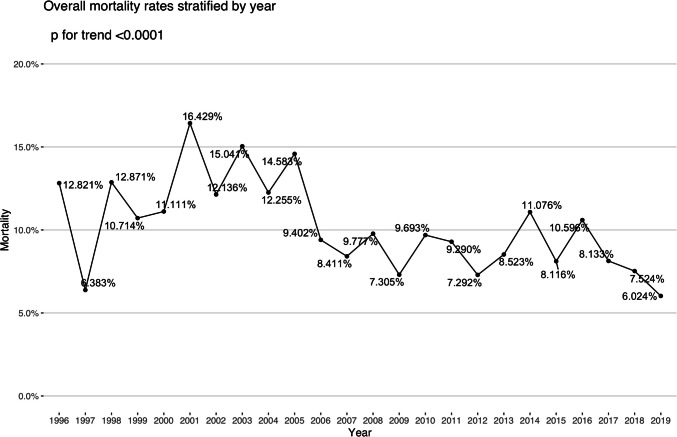
Year-wise mortality for re-operative AVR in the UK

**Fig. 2:**
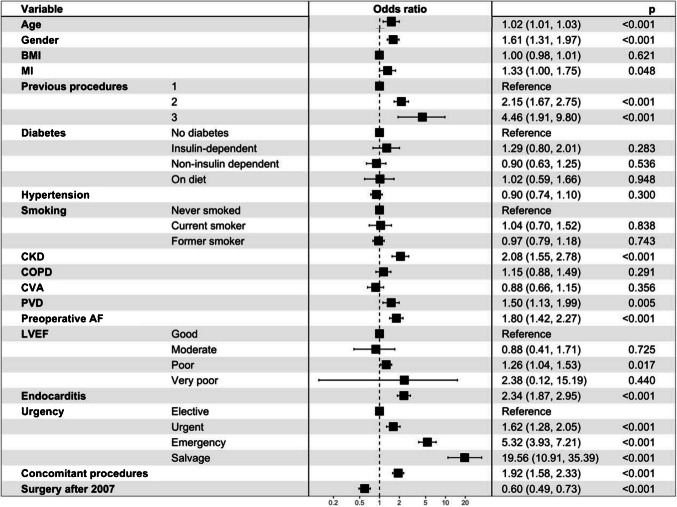
Forest plot showing the odds ratios (with 95% confidence intervals) of independent predictors of in-hospital mortality following re-operative AVR. Factors such as age, female gender, number of previous operations, urgency of procedure, presence of chronic kidney disease, peripheral vascular disease, preoperative atrial fibrillation, impaired LV function, and need for concomitant procedures were associated with increased mortality. Surgery performed after 2007 was associated with improved survival

**Fig. 3:**
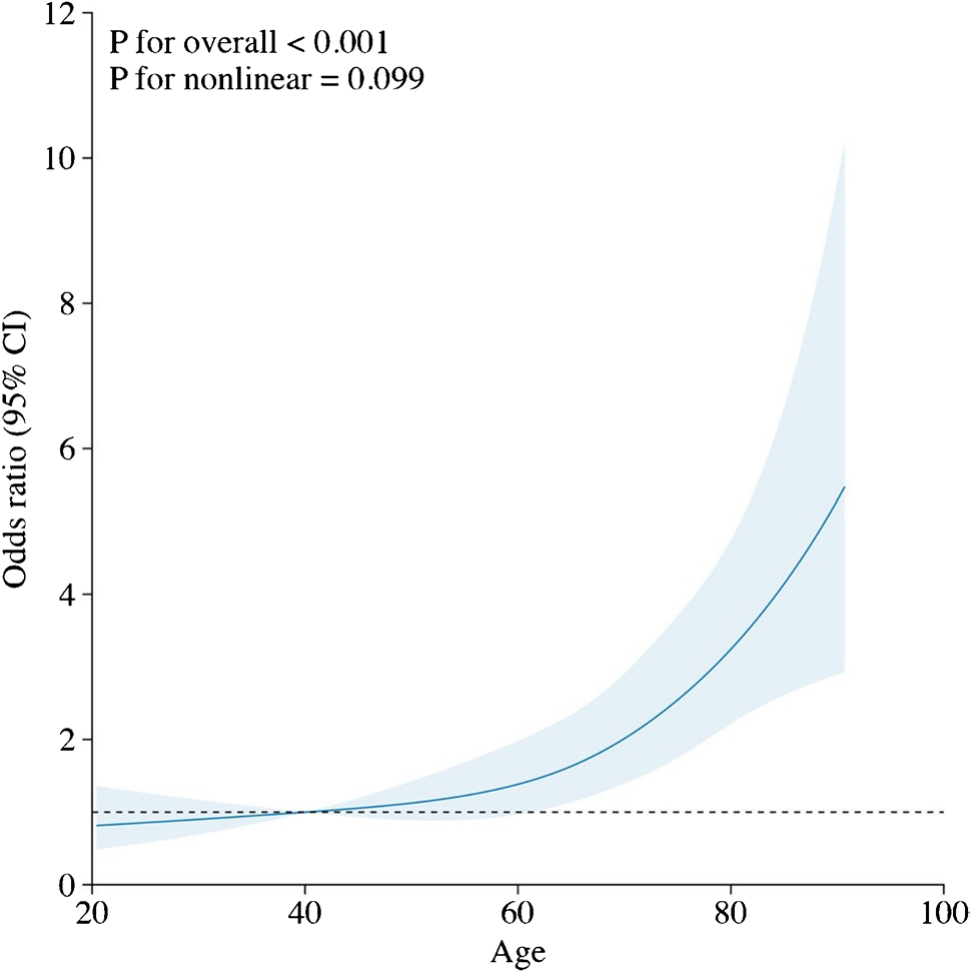
Restricted cubic spline plot illustrating the relationship between patient age and adjusted risk of in-hospital mortality following re-operative aortic valve surgery. The risk of mortality increases steadily beyond the age of 40 years, with a more pronounced rise seen after age 70 years. The plot is adjusted for other significant variables identified in the multivariable logistic regression model

The overall mortality in the cohort was 9.8% (599). The mortality rate for re-operative cases in elective procedures was 4.8% (166 out of 3,400). In patients undergoing urgent re-operation, the mortality increased to 11.8% (261 out of 2,208). For emergency cases, the mortality rate was significantly higher at 31.7% (137 out of 432), and it reached 50.7% (35 out of 69) in salvage cases (Table 1). The mortality associated with endocarditis was significantly higher at 18.8% (299) compared to 6.6% (300) in those without endocarditis (*p* < 0.001). Mortality rates showed a steady decline from 16.4% in 2001 to 6% in 2019 (Fig. 1). The length of hospital stay was significantly shorter among non-survivors (Table 3), with half the patients dying within four days of surgery. The stroke rate (6.5% vs. 1.7%) and need for postoperative dialysis (32% vs. 4.0%) were significantly more common in non-survivors than survivors. Return to operation theatre was required in 9% of cases, with re-exploration for bleeding accounting for 6.5% of these cases. Multivariate regression analysis showed that age, female gender, number of previous operations, presence of chronic kidney disease (CKD), peripheral vascular disease (PVD), pre-operative atrial fibrillation (AF), LV function, the urgency of operation and multiple procedures were independent predictors of adverse outcomes in these patients (Table 4). Salvage and emergency operations had the highest associated mortality risks while surgery performed in the latter half of the study period was protective against mortality (Fig. 2). The restricted cubic spline curve of age vs. mortality adjusted for the significant variables f have been plotted (Fig. 3). The risk is shown to start increasing beyond the age of 40 years with a dramatic increase beyond the age of 70 years.

**Table 3:** Postoperative outcomes

	Overall, *n* = 6,109	Survivors *n* = 5,510	Non-survivors *n* = 599	*p*-value
Length of hospital stay (days)	10 (7, 17)	10 (7, 17)	4 (1, 16)	< 0.001
Return to theatre	552 (9.0%)	409 (7.4%)	143 (24%)	< 0.001
Neurological Dysfunction				< 0.001
TIA	63 (1.0%)	52 (0.9%)	11 (1.8%)	
Stroke	135 (2.2%)	96 (1.7%)	39 (6.5%)	
Deep Sternal Wound Infection	30 (0.5%)	21 (0.4%)	9 (1.5%)	0.002
Post-operative Dialysis	413 (6.8%)	221 (4.0%)	192 (32%)	< 0.001
Post-operative IABP	75 (1.2%)	44 (0.8%)	31 (5.2%)	< 0.001

*TIA* Transient Ischaemic Attack, *IABP* Intra-Aortic Balloon Pump

**Table 4:** Multivariable logistic regression parsimonious set of predictors for mortality based on Akaike information criterion using 500 bootstrap datasets at alpha = 0.05

Variable	Significance (%)
Surgery after 2007	100.0
Age	100.0
No of Previous operations	100.0
Urgency	100.0
Gender	98.2
Concomitant procedures	98.0
Pre-op Chronic Kidney Disease	94.6
Endocarditis	90.8
Preoperative AF	80.4
LVEF	80.0

*LVEF* Left Ventricular Ejection Fraction

## Discussion

The overall mortality rate in patients undergoing re-operative AVR in the entire cohort was 9.8% and was 4.8% in elective reoperative AVR. Degenerative changes in the bioprosthetic valves constituted the commonest indication for re-operative surgery in nearly seven out of ten patients. Mechanical valves were more commonly explanted among non-survivors compared to survivors, and the predominant hemodynamic pathology was regurgitation in non-survivors. After adjustments, surgery after 2007, age, number of previous operations, urgency of operation, female gender, concomitant procedures, pre-operative CKD and endocarditis, were important predictors of outcomes. Preoperative AF and LV function at the time of operation also predicted mortality outcomes in these patients, but to a relatively lesser degree.

Reoperative surgery has been shown to have an independent odds ratio of 1.6 for mortality [21]. In addition, several other co-morbidities and operative factors further increase the risk of mortality after reoperative surgery. Age [22, 23], gender [22, 23], LV function- [14, 24], concomitant CABG [3, 6, 16, 17], emergency surgery [16, 17, 24], endocarditis [3–6], multiple previous operations [25, 26] have all been identified as risk factors for mortality during re-operative AVRs and have been confirmed in our study. In a previous report we have described that during the same time period, 40,858 primary isolated AVRs were carried out in the United Kingdom with a mortality of 1.9% for primary AVR and 3.1% of isolated reoperative AVR [27].

Urgency of the re-operative procedure was the most significant determinant of mortality in our study. Compared to elective procedures, the risk of mortality was 10-times higher during a salvage procedure and nearly 6-times higher during emergency re-operative surgery. Similar observations have been made by other studies, which showed a 10 to 16-fold increase in patients requiring emergency re-operative surgery [17, 24]. The clinical implication of this observation is the need for regular follow-up, which may identify a problem with the valve and minimize the need for emergency surgery, although this may not always be possible in certain cases, like endocarditis. Early reoperations have been associated with a mortality rate as low as 1%, comparable to that of first-time AVRs [16].

Pre-operative LV dysfunction was another important predictor of mortality in our study, with an OR of 1.3 for severe LV impairment and 1.6 for very severe LV impairment. Other studies examining the relationship between LV dysfunction and mortality have reported an even greater influence of LV dysfunction on mortality. The multicenter European RECORD (REdo Cardiac Operation Research Database) reported a greater than 8 times risk of mortality in the presence of LVEF < 30% [14]. Another study reported a nine-fold increase in mortality in the presence of impaired LV function [24].

Degenerative changes in bioprosthetic valves constituted the most frequent indication for AVR in our study, consistent with observations from other studies [28]. While mechanical valves were explanted more frequently among non-survivors compared to survivors (32% versus 26%), this relationship was significant only on univariate analysis and did not remain an independent predictor of mortality after adjustment.

Increased mortality in patients where re-operative surgery was indicated on mechanical valves has been shown by other researchers [3, 15, 18]. However, in our study, the crude mortality rate associated with explanted mechanical valves was 11.5%, slightly higher than the 9.2% observed for bioprosthetic valves. The observed differences in mortality are likely influenced by the clinical urgency of reoperations rather than the valve type itself. In our study, urgent, emergency, and salvage procedures were significantly more common in the mechanical valve group. After adjusting for procedural urgency, the type of explanted valve was no longer identified as an independent predictor of mortality. These findings emphasize that the urgency and nature of reoperation are key determinants of outcomes, rather than the type of valve explanted.

In our series, endocarditis was an etiology in 25.9% of the cases, which is similar to reports from other databases [3, 6, 14] and in keeping with isolated institutional reports published from the UK centers [3–6, 14]. While endocarditis can affect both biological and mechanical valves the incidence of endocarditis as an etiology for reoperations in bio-prosthetic valves seems to be lower [1, 18]. The incidence of endocarditis has also been reported to be higher in younger patients [18].

Multiple previous reoperations and concomitant surgery also appeared to independently influence mortality in our series. The effect of multiple reoperations has been assessed in few previous studies and the number of previous operations was found to be a moderately strong risk factor for mortality [16]. Another study has shown that the mortality with third re-intervention was 18.2%, compared to 7.3% in the first re-intervention on the aortic valve [25]. Need for concomitant CABG was also identified as a risk factor in several studies [3, 6, 16, 17]. An earlier study highlighted the consistent increase in mortality with the number of previous operations in the aortic position. While the first reoperation carried a mortality of 11%, it was 33% for third or more reoperations [26].

The cardiopulmonary bypass (CPB) time and cross clamp time were significantly longer among non-survivors, a reflection of these patients requiring more complex surgery and concomitant procedures. One in four patients among the non-survivors required two or more valves to be replaced during the reoperation and one-third of the patients had undergone concomitant CABG. Prolonged CPB time beyond 165 min has been shown to have moderate discriminatory value for in-hospital mortality [14].

In the early part of the study the mortality was higher, similar to reported by earlier studies [12, 26, 29], with subsequent improvement noted later. Studies in the last decade have reported mortality in re-operative surgery after previous aortic valve intervention in the range of 5% [1, 5, 14]. The mortality in the last year of our study period was lowest at 6%. The reduction in mortality likely reflects the cumulative impact of appropriate patient selection, advancements in surgical techniques, improved perfusion and anaesthetic strategies, and optimized perioperative care. Increased surgical familiarity with complex reoperative procedures—not only in the context of AVRs but across various areas of cardiac surgery—has likely contributed to better technical execution and intraoperative decision-making. Advancements in myocardial protection strategies, blood conservation strategies and infection control measures have also likely contributed to improved survival. Technological advancements leading to more precise hemodynamic monitoring, leading to early postoperative interventions, may also have contributed to the reduction in the incidence of complications. Our multivariate analysis confirms that procedures performed in the latter era were independently associated with lower mortality, suggesting that these systematic improvements translate into better patient outcomes.

Compared to some of the more contemporary series, the mortality in our series was relatively higher. This may be attributed to the fact that nearly one-third of the patients in our cohort had significantly impaired LV function, with an ejection fraction below 30%. In contrast, the study by Greco et al., which reported a mortality of 3.8% had only 15% of patients with impaired ejection fraction [5]. Similarly, the patients with LVEF < 30% comprised only 5.8% in the European RECORD study, which reported a mortality of 5.1% [14]. Moreover, the pathology necessitating reoperation also may have influenced the mortality. Comparison with other studies is limited, as our analysis specifically includes patients whose initial operation was an AVR, whereas other studies have included non-aortic valve procedures. Additionally, our cohort includes patients who may have undergone multiple concomitant procedures and had more than one previous operation on the aortic valve [5, 14].

### Limitations

The study was a retrospective study carried out on prospectively collected data and has the inherent limitations of a retrospective design. The outcome reported are limited to in-hospital data and linking this data to longer term survival data has not been possible. However, the study provides one of the most exhaustive reports on re-operative aortic valve surgery in the United Kingdom over the last 23 years.

## Conclusions

With improved management strategies, mortality in re-operative AVR continues to decrease, but still remains considerable. Degeneration of bio-prosthetic valves remain the commonest indication for re-operative surgery on the aortic valve. Emergency procedures carry a much higher mortality, and every attempt should be made to follow-up these patients closely and replace a failing valve under elective condition.

## Supplementary Information

Below is the link to the electronic supplementary material.

Supplementary file1 (MP4 15665 KB)

## Data Availability

The data underlying this article were provided by National Institute of Cardiovascular Outcomes Research (NICOR). Data can be shared on request to the corresponding author subject to permission from NICOR.
